# Examining the experiences of vulnerably housed patients visiting Kingston, Ontario’s emergency departments: a qualitative analysis

**DOI:** 10.1186/s12939-024-02217-0

**Published:** 2024-07-10

**Authors:** Lezhanska Anastasiya, Walker Melanie, Susan Bartels A, Fyfe Judy, Purkey Eva

**Affiliations:** 1 Department of Family Medicine, Queen’s University, 220 Bagot St, Kingston, ON K7L 3G2 Canada; 2 Department of Emergency Medicine, Queen’s University, 76 Stuart Street, Victory 3, Kingston, ON K7L 2V7 Canada; 3 Department of Public Health Sciences, Queen’s University, 62 Fifth Field Company Lane, Kingston, ON K7L 3N6 Canada; 4 St. Vincent De Paul Society of Kingston, 85 Stephen St, Kingston, ON K7K 2C5 Canada

**Keywords:** Homelessness, Vulnerably housed, Equity-deserving groups, Emergency medicine

## Abstract

**Introduction:**

Vulnerably housed individuals access emergency departments (EDs) more frequently than the general population. Despite Canada’s universal public health care system, vulnerably housed persons face structural barriers to care and experience discrimination from healthcare providers. This study examines how vulnerably housed persons perceive their experience of care in the ED and Urgent Care Center (UCC) in Kingston, Ontario and aims to develop strategies for improving care for this group.

**Methods:**

As part of a larger mixed-methods study, narratives were collected from participants attending the ED/UCC as well as community-based partner organizations, asking them to describe an experience of a recent ED visit (< 24 months). Participants could identify as members of up to three equity-deserving groups (EDGs) (for example homeless, part of an ethnic minority, having a disability, experiencing mental health issues). Coding and thematic analysis were completed for the experiences of participants who identified as being vulnerably housed (*n* = 171). Results were presented back to individuals with lived experience and service providers working with clients with unstable housing.

**Results:**

Participants reported judgement related to a past or presumed history of mental health or substance use and based on physical appearance. They also often felt unheard and that they were treated as less than human by healthcare providers. Lack of effective communication about the ED process, wait times, diagnosis, and treatment led to negative care experiences. Participants reported positive experiences when their autonomy in care-decision making was respected. Furthermore, having a patient-centered approach to care and addressing specific patient needs, identities and priorities led to positive care experiences.

**Conclusions:**

The ED care experiences of vulnerably housed persons may be improved through healthcare provider training related to trauma-informed and patient-centered care and communication strategies in the ED. Another potential strategy to improve care is to have advocates accompany vulnerably housed persons to the ED. Finally, improving access to primary care may lead to reduced ED visits and better longitudinal care for vulnerably housed persons.

## Background

Homelessness is a major risk to health and conversely, poor health often contributes to the many causes of homelessness [1]. Despite Canada’s universal public health care system, vulnerably housed persons face structural barriers to healthcare including access and financial difficulties (i.e. costs of medications, medical supplies, transportation to appointments, etc.), as well as having to frequently prioritize obtaining shelter and other basic needs over healthcare [2]. Additionally, vulnerably housed individuals experience discrimination from healthcare providers and may avoid seeking care due to mistrust or to previous negative care experiences [2]. Research has shown that unhoused patients experience longer wait times in Emergency Departments (EDs) than housed patients, particularly in the urgent and emergent triage categories [3, 4]. Another study has shown that unhoused American veterans visiting the ED feel they have no input into care decisions and that this leads to mistrust of the system and of healthcare providers [5]. Evidently vulnerably housed patients have unique experiences that impact their perception of care, their utilization of the ED, and their overall health status.

At least 235,000 Canadians experience homelessness every year and, on any given night, 35,000 Canadians are homeless [6]. Furthermore, it is estimated that many more individuals are experiencing hidden homelessness, meaning they avoid staying in shelters or living on the street by “couch surfing” or other means [7]. In 2018, approximately 15% of Canadians responsible for housing decisions in their household reported having experienced hidden homelessness [8]. Research has shown that the number of people experiencing homelessness is increasing in Ontario [9, 10]. In recent years, the demographics of the vulnerably housed population in Canada have shifted to include higher proportions of youth, Indigenous people, women, immigrants, veterans, older adults, and people from rural communities [11, 12]. Those who are vulnerably housed face an intersection of social, physical, and psychological factors that significantly increase morbidity and mortality [2, 11]. On average, life expectancy for vulnerably housed persons is estimated to be between 42 and 52 years [13]. Individuals living outside or in shelters have an increased risk of communicable disease, violence, food insecurity, and environmental exposures [1, 12]. These risks can lead to high ED use [2, 14].

The number of unhoused patients accessing EDs in Ontario is increasing [10]. Research has shown that the number of unique vulnerably housed individuals presenting to an Ontario ED increased from 4,203 in 2010 to 9,350 in 2017 [10]. Given this increase, it becomes imperative to understand the experiences of unhoused people within the healthcare system to allow the system to make the necessary changes in order to ensure that these individuals are able to access appropriate, safe care.

This analysis examines the experiences of vulnerably housed persons accessing the ED and Urgent Care Center (UCC) in the medium sized Canadian city of Kingston, Ontario. The objective of this study was to better understand how vulnerably housed patients perceive their experience of healthcare, and consequently, to determine areas for improvement in the care of this group.

## Methods

### Data collection

Data were derived from a larger 2021 cross-sectional study investigating Kingston ED care experiences among members of equity-deserving groups (EDGs), for example homeless, members of ethnic minority groups, having a disability, experiencing mental health issues, among others [15]. Equity deserving groups are those who have traditionally experienced disadvantage or marginalization in society or, in this case, within the healthcare system.

Using a mixed methods approach, participants were given a prompt and asked to share a narrative about a past ED/UCC experience. Example of prompts include sharing a story about your best or worst experience in the ED. Following this, participants answered a series of pre-programmed questions both about their own sociodemographic information and about their experience of care. Participants were able to self identify as a member of up to three EDGs. An example of a question related to experiences of care include: “during the emergency room visit the patient’s personal situation, identity or culture received far too much or far too little attention” (Likert scale). Through this process, participants, who have more information about their story that the simple narrative that is shared, can provide additional “analysis” in the form of answers to prompted questions, thereby allowing investigators to have a more nuanced understanding of the participant’s perception of their experience. While mixed methods data was collected as part of the larger study, the data used for this analysis is purely qualitative.

Spryng.io, a narrative capture tool, was used to audio-record the participant’s story and transcribe it into written format, thus producing the qualitative data. Spryng.io also records the participants responses to additional questions. The survey took an average of 15 min to complete.

In this study, the terms “vulnerably housed”, “unhoused” and “homeless” included those individuals who are living in temporary or unstable accommodations, outdoors, or in shelters.

### Setting

Data were collected from June to August 2021 in Kingston, Ontario. Kingston has an estimated total population of 172,546 as of 2021 [16]. According to the 2021 Urban Kingston Point-in-Time Count, the total number of people experiencing homelessness in Kingston increased by almost 27% since 2018 [17]. These numbers likely underrepresent the true nature of the problem as they account only for people staying in shelters, institutions, or public spaces on the night of the count, and not for those experiencing hidden homelessness. Data collection was carried out at the Kingston Health Sciences Centre (KHSC) ED, a single urban ED with annual patient volumes of 57,648, and at Hotel Dieu UCC, a single urban UCC with annual patient volumes of 37,708 [15], as well as at community-based partner organizations.

Trained research assistants facilitated survey completion. They collected information Monday to Friday from 9AM to 9PM at both sites. Data were also collected from clients visiting community-based partner organizations including: the Kingston Youth Shelter (local shelter providing emergency shelter, transitional housing, and family mediation for youth from age 16–24); One Roof Kingston (youth hub providing wrap around services to vulnerable youth in Kingston); Kingston Street Health Centre (organization providing wrap around primary care and substance use care for people facing barriers to accessing the mainstream health services); and St. Vincent de Paul Society of Kingston (providing emergency food, clothing, household supplies and programming for low income people in Kingston), among others. This was done to capture the perspectives of individuals not actively seeking care in the ED during the study period, potentially as a result of previous negative experiences.

### Participants

**Fig. 1:**
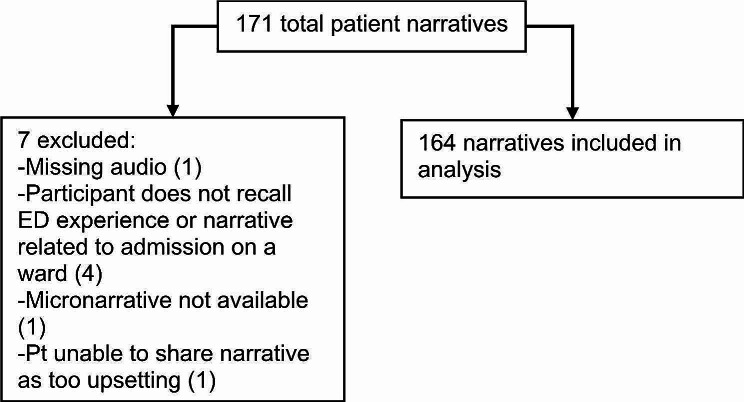
Flow diagram of included vs. excluded participant narratives

Eligible participants for the overarching study included anyone aged 16 years and older who had visited the ED or UCC within the previous 24 months, either as a patient or accompanying a patient. Participants had to be able to give informed consent and complete the survey in English. Research assistants did not approach patients in the ED/UCC who were medically unstable, did not possess the capacity to provide informed consent, or were aggressive towards staff. Of the overarching study 2114 total participants, 171 self identified as being homeless or vulnerably housed and were retained for this study. 7 narratives were excluded (see Fig. 1) and 164 narratives were included in the analysis.

### Data analysis

Iterative reviews and coding of the data were performed by the first author (AL) with regular review by a senior faculty member experienced in qualitative research (EP). NVIVO software [18] was used to support data management and organization. Thematic analysis was conducted using an inductive approach based in grounded theory, whereby important themes emerge from progressive coding and comparison of the narratives [19]. In addition to other coding, aspects of patient narratives were coded to either a positive, negative, or neutral experience. A given narrative might contain both positive, neutral, and negative experiences. As the main themes emerged in the analysis, each theme was supported by narrative content coded to positive and negative experiences.

### Focus groups

Findings from the thematic analysis were presented at two community focus group discussions: one with individuals with lived experience of homelessness and one with service providers who work with equity-deserving groups on a regular basis, helping them navigate systems and receive appropriate services. Participants of focus group discussions were affiliated with the Kingston Street Health Centre, St. Vincent de Paul Society of Kingston, and/or the Integrated Care Hub (low barrier shelter and safe injection facility). These discussions assessed the validity of our results and provided further insight into each of the themes as well as future recommendations. The discussion comments were summarized in a written document during the focus group and then reviewed by the researchers. All comments were consistent with the findings based on participant narratives and validated the findings.

### Ethical considerations

The study, along with the data collection tools, was co-designed in collaboration with members of EDGs and community-based organizations including Indigenous partners led by the Indigenous data governance and research principles of Ownership, Control, Access, and Possession (OCAP) [20].

Data collection was anonymous, and no identifying information was collected from participants. Informed consent was obtained prior to completion of the survey. Participants were provided with a $5 coffee gift card as a token of appreciation. The study was approved by the Queen’s University Health Sciences and Affiliated Teaching Hospitals Research Ethics Board.

## Results

From a total of 2114 collected narratives, 171 were provided by participants who identified as being vulnerably housed. These 171 narratives were read in their entirety by two researchers (AL and EP). Seven narratives were excluded (Fig. 1), leaving 164 for thematic analysis and coding.

Table 1 summarizes the characteristics of the participants and the ED visit. Of the total narratives provided by persons identifying as vulnerably housed, 89% were primary accounts where the person in the narrative was also the patient. The majority of participants were aged 26–45 years (38%) and more men participated (57%) than other genders. The predominant ethnicity of participants was white/European (47%). A greater proportion of experiences took place at the Kingston General Hospital ED (77%) than the UCC and more experiences had occurred within the previous 0–6 months (35%) than longer ago. Of those who chose to disclose, more participants had accessed the ED 1–3 times in the preceding 24 months (32%), although data is missing for 33% of participants.

During analysis, four major themes emerged. Each theme was illustrated by both positive and negative examples and will be discussed from both perspectives. The four themes were (1) experiences and consequences of stigma and judgement; (2) experiences of dehumanization; (3) communication in the ED; and (4) patient-centered care. These themes were consistent with feedback from both the vulnerably housed focus group and service provider focus group.

**Table 1:** Participants and ED visits characteristics

Variable	*N* (%)
**Who was the patient in the story?**	
It was me	153 (89%)
It was someone else in my family	9 (5%)
It was a friend	5 (3%)
Other	3 (2%)
It was a person for whom I am a caregiver (paid or volunteer)	1 (1%)
Total	171 (100%)
**Patient age**	
26–45 years of age	65 (38%)
46–65 years of age	39 (23%)
18–25 years of age	11 (6%)
Greater than 65 years of age	6 (4%)
Less than 18 years of age	4 (2%)
Missing	46 (27%)
Total	171 (100%)
**Patient gender**	
Man	98 (57%)
Woman	63 (37%)
Non-binary	6 (4%)
Missing	4 (2%)
Total	171 (100%)
**Patient Ethnicity**	
White /European	80 (47%)
Indigenous	22 (13%)
Other (Latin American, South, Southeast, or West Asian, Filipino, Other)	10 (6%)
One or more ethnicity	6 (4%)
Black	3 (2%)
Missing	50 (29%)
Total	171 (100%)
**How long ago was the shared ED visit**	
0–6 months	60 (35%)
7–12 months	25 (15%)
13–18 months	17 (10%)
19–24 months	15 (9%)
More than 24 months	7 (4%)
Missing	47 (27%)
Total	171 (100%)
**Number of visits to the ED in the preceding 24 months**	
1–3 times	55 (32%)
Greater than or equal to 4 times	45 (26%)
Did not access care in the emergency	15 (9%)
Missing	56 (33%)
Total	171 (100%)
**Where did the shared narrative take place**	
Kingston General Hospital Emergency Room	131 (77%)
Hotel Dieu Hospital Urgent Care Centre	40 (23%)
Total	171 (100%)

### Theme 1: experiences and consequences of stigma and judgement

There were numerous negative experiences relating to stigma and judgement (Table 2). Participants described experiences where healthcare providers would focus on their history of past mental health or substance use instead of asking about their current reason for presenting to the ED. Furthermore, participants described healthcare providers assuming that there was a history of substance use or mental health leading to the ED visit, even when the patient had no such history. Participants also felt there was a lack of appropriate symptom management, especially pain, due to assumptions about drug use or addiction. In addition, participants discussed judgement based on clothing or appearance.

Insight from the service provider focus group revealed that there is a frequent phenomenon of vulnerably housed persons being discharged from the ED without a physical exam or proper care and then having to return with an advocate to ensure the proper treatment was provided. Service providers reported having seen cases where this led to very serious illness being missed and getting worse as a result.

**Table 2:** Experiences of stigma and judgement – negative participant quotes

*“I once was at KGH and the security and nurses at triage judged me by my look assuming I am an addict when I was actually sober for couple months by then. […] Although I was in for an infection on my finger, they automatically assumed I was there for something else asking tons of questions related to my mental concerns and history of substance use.” (Male, age 26–45)*
*“They just assumed that she was there for pain medication and sent her home and didn’t even bother looking.”(Female, age 26–45)*
*“So no, any time I have been to [name of ED] I have never ever had a good experience. It has been the same way every time. Because of the way I look they automatically judge me. And that means I don’t get the best care.” (Female, age 26–45)*

Participants also provided positive examples related to not having the anticipated experiences of stigma and judgement (Table 3). They described receiving sensitive care from healthcare providers who were conscientious of their personal situation and showed respect toward them. Participants reported being often surprised by this care.

**Table 3:** Experiences of stigma and judgement – positive participant quotes

*“And they were very, very helpful. And not judgemental like I have had issues before in the hospital. But no, I was treated you know with a lot of respect, um, from pretty much everyone even security.” (Female, age 26–45)*
*“I was battling addiction and other problems severely in the past and during my stages of the vicious cycle I was living I was very sick and found the [name of ED] to be a lotta help for me mentally and physically along with my emotional side of things but during my stay or visit I felt a type of help I couldn’t get no where else so I firmly stand by the system they provided me here.” (Male, age 18–25)*
*“The treatment was really good. I was expecting not a good quality service or bad attitude but staff were nice.” (Male, age not disclosed)*

### Theme 2: experiences of dehumanization

Participants expressed experiences of being treated as less than human by staff in the ED (Table 4). They felt they received less respect and were considered less important than other patients or staff members. They also described feeling ignored and that their concerns were not addressed properly as a result.

**Table 4:** Experiences of dehumanization – negative participant quotes

*“They seemed more like fluffing me off like I was nothing. […] And they just kind of shuffled me through as if I didn’t mean to be anyone or a person or anyone that of importance.”(Female, age 26–45)*
*“They kind of were feeling like I felt that they were undermining me quite a bit. Um, and undermining the symptoms that I felt and kind of just looking at me like I was, I don’t know lesser than them.” (Male, age 18–25)*
*“I felt like the doctors were of the opinion that I was not priority because I was not likely to be a contributing member of society.”(Male, age 26–45)*

However, participants also shared experiences of being listened to and valued and expressed how much this impacted their experience in a positive manner (Table 5).

**Table 5:** Experiences of dehumanization – positive participant quotes

*“I have to say that the team was outstanding. It was nice to be recognized that it was a legitimate problem, and I can’t say enough about that group of people.” (Female, age 46–65)*
*“I was so impressed with the staff and how they dealt with the wide range of people. Some of whom were angry and crass. Left thinking that the staff were so professional and kind even when they were faced with some difficult behaviour. I am so grateful for how well my mother was treated but also impressed with how even the disruptive patience were treated with respect and kindness.” (Female, age 65 or older)*

### Theme 3: communication in the ED

The theme of communication in the ED encompassed a range of experiences. Some of the predominant negative experiences (Table 6) involved a lack of patient involvement in decision-making. Participants described poor communication about diagnosis or treatment. They also reported frustration at long wait times, and often felt it was related to healthcare provider bias toward them. Furthermore, participants described cases of staff behaving rudely towards them when they expressed frustration at the wait times instead of communicating about the process and explaining the reason behind the wait. Service providers reflected that one of the systemic problems is the strained healthcare system, leading to overworked and busy healthcare providers who do not have the time to provide detailed explanations and address these issues adequately.

Participants also felt frustrated at having to repeat their story multiple times to different providers and learners. This can be especially harmful if individuals are asked about traumatic experiences and sensitive health details, particularly among vulnerably housed patients who may have higher rates of mental health issues, substance use disorders and trauma. Moreover, there were several experiences related to a lack of privacy in the ED. Therefore, sensitive topics were being discussed where other staff and patients could overhear.

**Table 6:** Communication in the ED - negative participant quotes

*“My care wasn’t my personal business. Somebody else involved themselves my care. And when they did they thought that they would take it upon themselves to make decisions for me.” (Male, age 46–65)*
*“I went to the hospital because I swallowed some drugs and they were really not explaining anything to me. Then I signed papers from the police that I didn’t know I signed and then I fell asleep and woke up and they just let me go.”(Female, age 26–45)*
*“One thing I remembered was that I was left in the room by myself without having any staff informing me which procedures I was in.” (Female, age 26–45)*

By contrast, participants shared positive experiences when staff respected patient autonomy in decision-making and took time to explain topics and answer questions (Table 7).

**Table 7:** Communication in the ED - positive participant quotes

*“The staff took time to explain that the drug could been mixed in with weed I smoked as some people use the same scales and it gets cross-contaminated. I remembered that friend who shared weed with me is a heavy fentanyl user so it made sense. I was advised to be more careful with any substances and given a narcan kit. I was expecting a bad treatment which often comes along with overdoses, in my past experiences I was treated badly due to my addiction struggles. But in [name of ED] I felt cared for, not judged and treated fairly.” (Female, age 26–45)*
*“The staff were very good and helpful with my situation. Was given instruction on how to get through the problem. Took 8 weeks to heal. But got through it even though it was difficult.”(Male, age 46–65)*
*“But the doctors there were really nice. Like there was a doctor there that told me you know I could slice it open if you want to get it quicker, but you are in so much pain I can barely touch it. And he specified that it was up to you. And I was like oh god no, no more pain. It’s fine. And you know, I just relaxed.” (Female, age 26–45)*

### Theme 4: patient-centered care

Among the shared negative experiences, participants discussed how healthcare providers did not pay attention to their specific needs, priorities, or identity and that this impacted care significantly (Table 8). Vulnerably housed persons have specific needs and barriers, including financial difficulties and often no access to primary care. This may limit the ability to follow discharge instructions including filling expensive prescriptions or securing supplies or follow up and should be considered in the ED prior to discharge.

Participants also shared frustration at being asked for their address repeatedly when they did not have one or did not want to share. This can be especially harmful as patients experience stigma when labelled as homeless. Furthermore, participants were also asked for identification or a provincial health insurance card when they did not have one as a consequence of unstable housing. Finally, security was called frequently to attend to participants, often without explanation and in the absence of violent behaviour. This would often lead to participants feeling unsafe and targeted.

**Table 8:** Patient-centered care - negative participant quotes

*“And after I was sick, um, they kind of just kicked me out right away. They didn’t really give me a chance to recover. So, then I was homeless at that time. So I was, I was forced to be on the street, and I was sick, and I had nowhere to go. And it was the middle of winter too.” (Male, age 18–25)*
*“I had to sit close to the security guard booth because they told me so which made me really uncomfortable and humiliating.” (Male, age 26–45)*
*“I don’t understand why I need to provide my address when I don’t have one. I don’t have stable housing at the time I went to emergency room and was staying at [name of] shelter […] So I provided [name of] shelter address and nurses told me to use my real address. When I had a quick argument with a nurse at triage, she called security to oppress me whenever I tried to express my frustration. It can be sometimes terrifying to have big security guys so close to me especially after couple bad instances with securities.” (Female, age 26–45)*

However, when their identity and specific needs were addressed, participants expressed improvement in their perception of the experience and their care as a whole (Table 9).

**Table 9:** Patient-centered care - positive participant quotes

*“I arrived at the hospital. I didn’t have any ID. They were extremely nice. I was really, really upset. I didn’t know where I was going or nothing. They did everything for me. They got me a bus pass. They sent me to where I should go for help. And I just thought it was great. And I appreciate all of the help that they did for me.”(Female, age 46–65)*
*“And then I am homeless and no income. And they were really nice to give me some supplies, you know to take back. And they asked me if everything is all right being homeless. And if there’s anything that they can help with. And do you want me to contact any organization or anything to help me out. Do I have a place to stay at night? And they were very, very helpful.” (Female, age 26–45)*

## Discussion

In this qualitative thematic analysis, we examine 164 ED patient care narratives to better understand how vulnerably housed persons perceive their ED care. Key findings include patient experiences of stigma, judgement and dehumanization as patients report healthcare providers making assumptions about mental health and substance use and treating patients as lesser than themselves. However, there are also many positive experiences which illustrate how ED care is improved when no assumptions are made and patients are treated with respect. In addition, our findings show the value of good communication and including patients in care decision-making. Finally, adopting a patient-centered approach to care, and taking into consideration the specific needs, identity and priorities of vulnerably housed patients, is important for improving their care experiences.

Literature shows that vulnerably housed persons report feeling judged and disrespected by healthcare providers, especially with regards to assumptions about substance use [21, 22]. Additionally, unhoused individuals often feel ignored and separate from society and dehumanized by others [23, 24]. This population strives to feel heard and cared for and are very aware of dismissive behaviour from healthcare providers [24]. Negative, biased treatment is a major factor explaining why vulnerably housed persons resist seeking care [25]. Receiving respectful, trauma informed treatment may help facilitate trust which is valued among vulnerably housed individuals and can lead to a higher likelihood of seeking care and adhering to recommended treatment [26], thus improving health outcomes for a group with higher burden of morbidity and mortality.

This study demonstrated that having autonomy in decision-making and control over their care is especially important for unhoused patients. Vulnerably housed persons often lack agency in many areas of their life, particularly in their interfaces with services or people in a position of relative authority. Taking the choice away leads to a worse care experience and may increase the risk that they will not return to care when needed [27]. Research has shown that sharing information about care and involving vulnerably housed patients in treatment decision-making alleviated anxiety, fear, and feelings of isolation in the ED, and demonstrated to patients that healthcare providers are tailoring plans to their individual needs [28]. One study demonstrated that vulnerably housed patients welcome questions from ED healthcare providers related to housing status and resources [29]. However, our participants expressed concerns about feeling judged, misunderstood and a lack of privacy with these conversations. Therefore, a strengths based and patient-centered approach is needed to help patients feel comfortable in sharing their housing status and to provide the best care for their individual social needs.

Communication during the experience of care is also important. Study participants felt that frequent touchpoints from healthcare providers during the visit would significantly improve the experience. Patients also feel ignored, insecure, and frustrated at long wait times [28]. Frequently they may assume that nothing is being done for them during those times because of bias related to their homeless status. Therefore, it is key for healthcare providers to communicate about personal or systems level reasons for long wait times, and to check in regularly. The time from triage to getting into the ED is critical as this is when most patients decide to leave or stay. One study showed that nearly one in five (18%) unhoused patients leave the ED prior to being assessed by a physician [30]. Healthcare providers offering explanations of triage, prioritization of care based on severity of the condition, and reasons for delay in care can help vulnerably housed patients understand that the wait is not a result of stigmatization but rather the nature of the system [23]. Moreover, vulnerably housed individuals expressed in our focus group discussion that having estimated wait times posted throughout waiting areas would alleviate anxiety and prevent them from leaving without being seen by the doctor. In addition, there is an opportunity to provide support to unhoused patients while they are waiting in the ED, for example through screening assessments of social support needs and early referrals as appropriate [30].

As mentioned above, this study also highlighted challenges to patient privacy in the ED. Specifically, healthcare providers asked patients about sensitive information where others could hear, including requiring disclosure of address or housing status. Although in a busy and crowded ED this can sometimes be challenging to address, healthcare providers should make every effort to ensure patient confidentiality where possible. This is particularly important when discussing mental health, substance use, and any history of trauma. Participants also expressed frustration at hearing healthcare providers talking about them with other providers, especially in a derogatory manner. Conversations pertaining to patients outside of the clinical encounter should be limited to relevant medical care only and should be held in private wherever possible.

### Recommendations

In addition to the recommendations made above, two others stand out worthy of mention.

First, focus group respondents articulated the importance of having an advocate in the ED who can support the patient and act as a bridge between the patient and health or social service supports in the community. Options for this can include friends or family, but sometimes these are not available to support unhoused individuals. Other options include increasing the availability of case management, where one contact person guides the patient through ED care, inpatient stays, and community appointments [31]. This approach has been shown to improve social and clinical outcomes and reduce ED costs [32], improve housing stability, reduce substance use, and improve connection to primary care for clients [33, 34]. Finally, having a peer support network accessible in the ED with lived experience of homelessness or other aspects of vulnerability may be beneficial. The presence of a companion has been shown to reduce isolation and improve healthcare experiences [26, 28].

Second, vulnerably housed patients value care that is consistent and provided by a healthcare provider who knows their social and medical history [22, 35]. Improving access to primary care for vulnerably housed patients would reduce their ED use and provide regular, longitudinal care with the same provider to aid in building trust. This is a difficult problem to solve however, due to the gap in access to primary care broadly in Canada, and specifically for this population. Two Canadian studies respectively demonstrated that only 43% [36] and 56% [37] of homeless persons have a primary care provider.

In summary, recommendations for improved care include, but are not limited to:

1. Improving mandatory, paid staff training in trauma informed care in order to minimize true and perceived experiences of judgement, stigma and disrespect of people experiencing homelessness.
2. Enhancing care that prioritizes patient centered individualized care and patient autonomy.
3. Creating systems to inform patients about the process of care, including wait times, to enhance understanding and decrease anxiety throughout the care process.
4. Ensuring to the greatest extent possible that patient privacy in maintained, particularly in ED settings which are often crowded and in which patients can overhear discussions.
5. Ensuring that wherever possible patients are allowed to have a companion or advocate with them in the ED, and that if they do not have their own, the ED can provide them with a peer support worker or case manager who can fulfil this role.
6. From a health systems approach, decreasing provider burnout which in turn enables them to provide more compassionate, trauma informed care; increasing funding for case managers; and increasing funding and access to family physicians who can provide consistent continuity of care may both decrease utilization of emergency services and enhance patient experience throughout the system.

### Strengths and limitations

Patients in the ED may be experiencing a variety of strong emotions related to their particular presentation and to the environment which may influence the content of their chosen narratives. Therefore, narratives collected in the ED may not be reflective of every experience. Additionally, the results are not generalizable given that the study was conducted in a single center and used a convenience sample, though the literature does support the identified themes. Individuals who were aggressive with staff, medically unstable or not able to provide informed consent were not included in the study and therefore their experiences were not captured. As well, individuals presenting to the ED outside of study hours and those who do not speak English may have different care experiences. Moreover, although participants were approached at community-based partner organizations, vulnerably housed persons who do not access these services were not represented in the study. Furthermore, thematic analysis was conducted by researchers who are not members of this equity-deserving group. To mitigate this limitation, we presented results to service providers and individuals with lived experience of homelessness. Insights received at this focus group aligned with conclusions drawn from the coded data and no additional themes were identified which adds reassurance to the validity of our findings.

The strengths of this study include having access to vulnerable groups that are often difficult to capture in research. This work was co-designed in collaboration with members of EDGs and community-based organizations including Indigenous partners. Participants were gathered from the hospitals as well as community-based partner organizations including the Kingston Youth Shelter, Home Based Housing, Kingston Street Health Center, and the Integrated Care Hub, among others. Finally, results were reviewed and discussed with members of EDGs and service providers working with equity-deserving individuals. Therefore, this work was embedded in the very community it aims to benefit.

## Conclusion

Our study highlighted the experiences of care and areas for possible improvement in care for people with a lived experience of homelessness. These include patients’ experiences of stigma and judgement, the importance of autonomy and patient centered care, and the need for privacy, and for improved communication about care and the process of care. Broadly, an approach to healthcare delivery that is trauma informed and focussed on enhancing health equity for all patients is needed. While some of the required interventions must occur at the individual provider level, many are systemic, related to funding priorities and other strains on the healthcare system. At a moment in time when Canadians are struggling with housing insecurity, mental health challenges and an opioid epidemic, it becomes all the more essential that the healthcare system be given the material and human resources to be able to support the most vulnerable members of society in ways that provide them with safe and accessible healthcare.

## Data Availability

All quantitative data, survey tools and interview guides are available from the authors upon reasonable request.

## Abbreviations

EDG: Equity-deserving group
ED: Emergency department
UCC: Urgent care center
KHSC: Kingston health sciences center
OCAP: Ownership control access, and possession
